# Supplementary figures and images for: Multi-color dSTORM microscopy in Hormad1-/- spermatocytes reveals alterations in meiotic recombination intermediates and synaptonemal complex structure
Source: PLoS Genet. 2022 Jul 20;18(7):e1010046. doi: 10.1371/journal.pgen.1010046 (PMC9342782; doi:10.1371/journal.pgen.1010046)

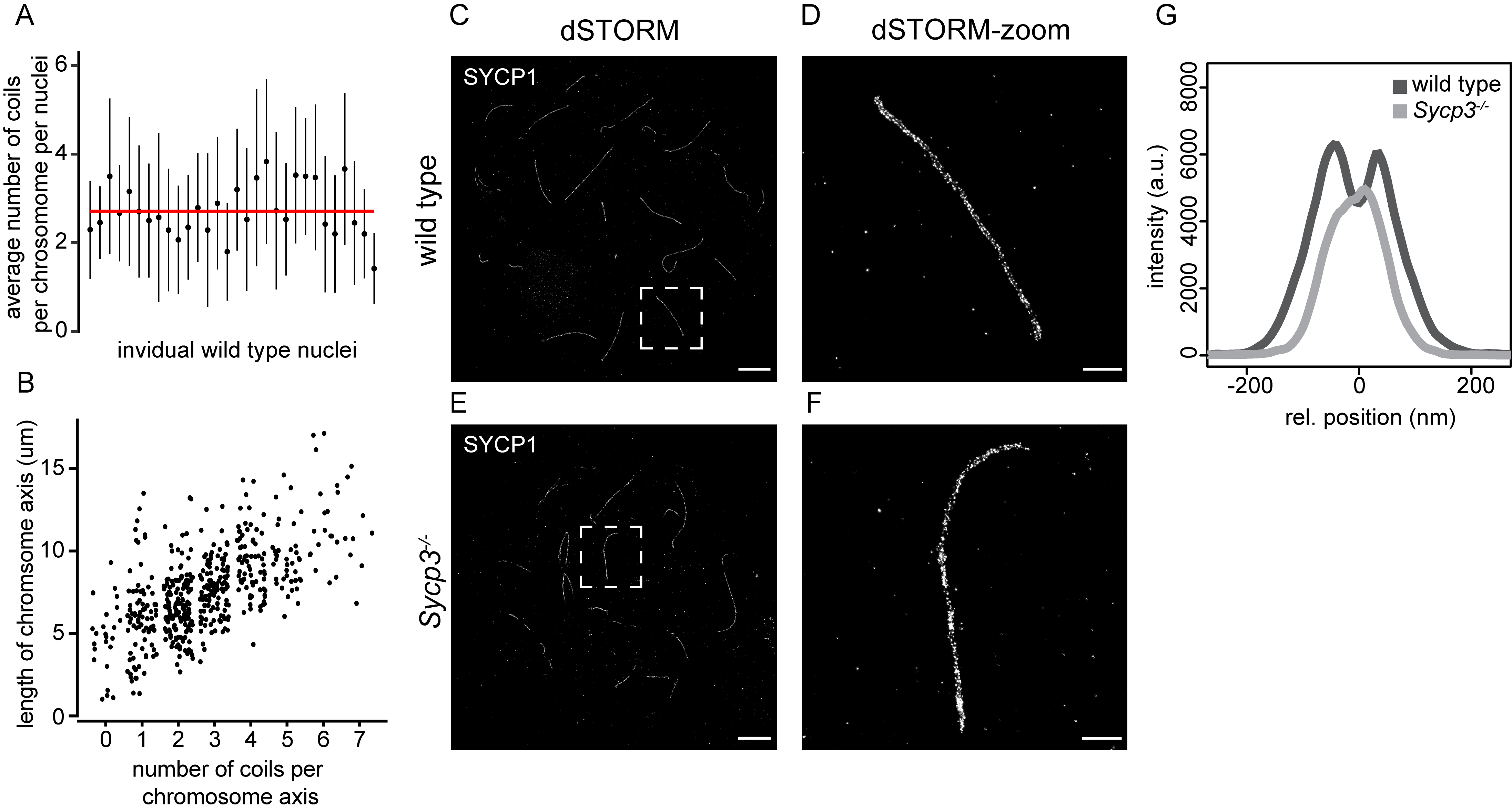

Supplement: S8 Fig — A) Plot of number of coils per chromosome averaged per nucleus. Median is indicated in red. B) Dotplot showing the relation between the number of coils per chromosome axes and the length of the chromosome axes. C) dSTORM and D) close-up of boxed region shown in C) image of wild type mid-zygotene nucleus immunostained for SYCP1 (white). E) dSTORM and F) close-up of boxed region shown in E) image of Sycp3-/- pachytene-like nucleus of immunostained for SYCP1 (white). G) Average intensity-profiles of SYCP1 at synapsed chromosomal axes for both wild type and Sycp3-/- (each consist of 21 profiles (3 regions per nuclei and 7 nuclei in total) for both genotypes). Scale bar represents 5 μm (C,E) and 1 μm (D,F). (TIF) [file pgen.1010046.s008.tif]

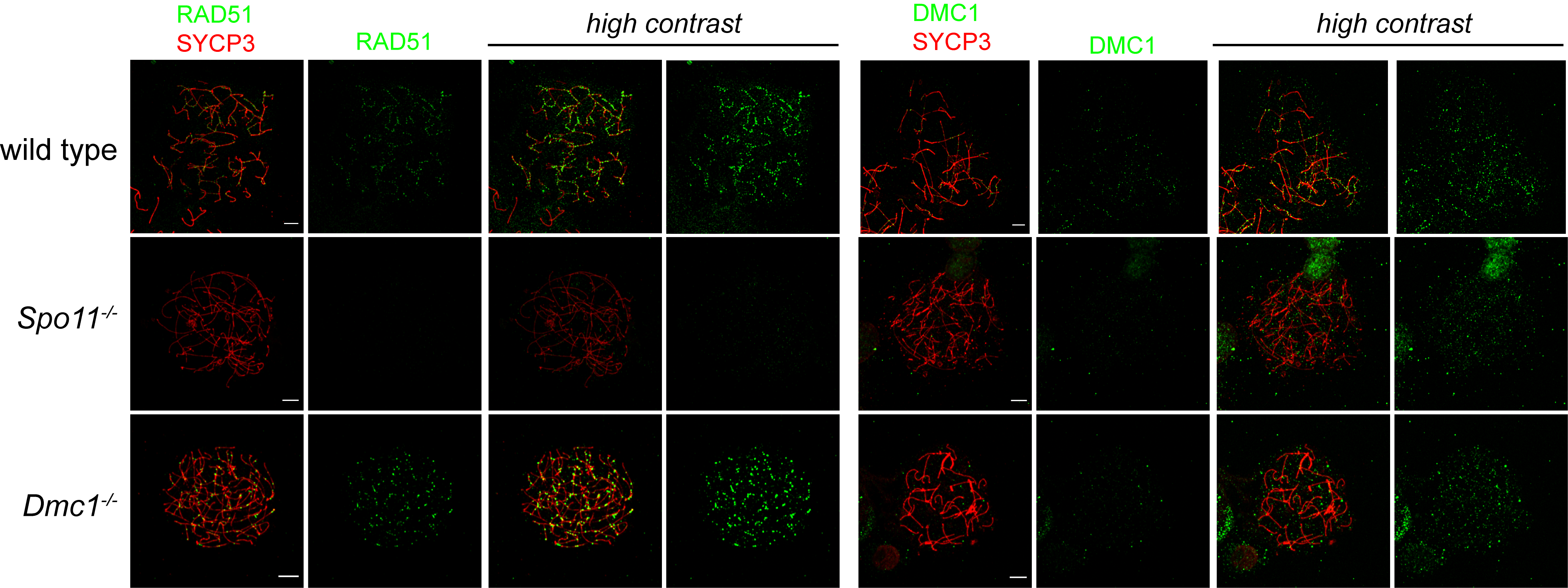

Supplement: S9 Fig — Confocal images of wild type, Spo11-/- and Dmc1-/- zygotene(-like) nuclei immunostained for SYCP3 (red) and RAD51 (green) (left) and SYCP3 (red) and DMC1 (green). Also images with a higher contrast are shown for each recombinase staining. Scale bars represent 5 μm. (TIF) [file pgen.1010046.s009.tif]

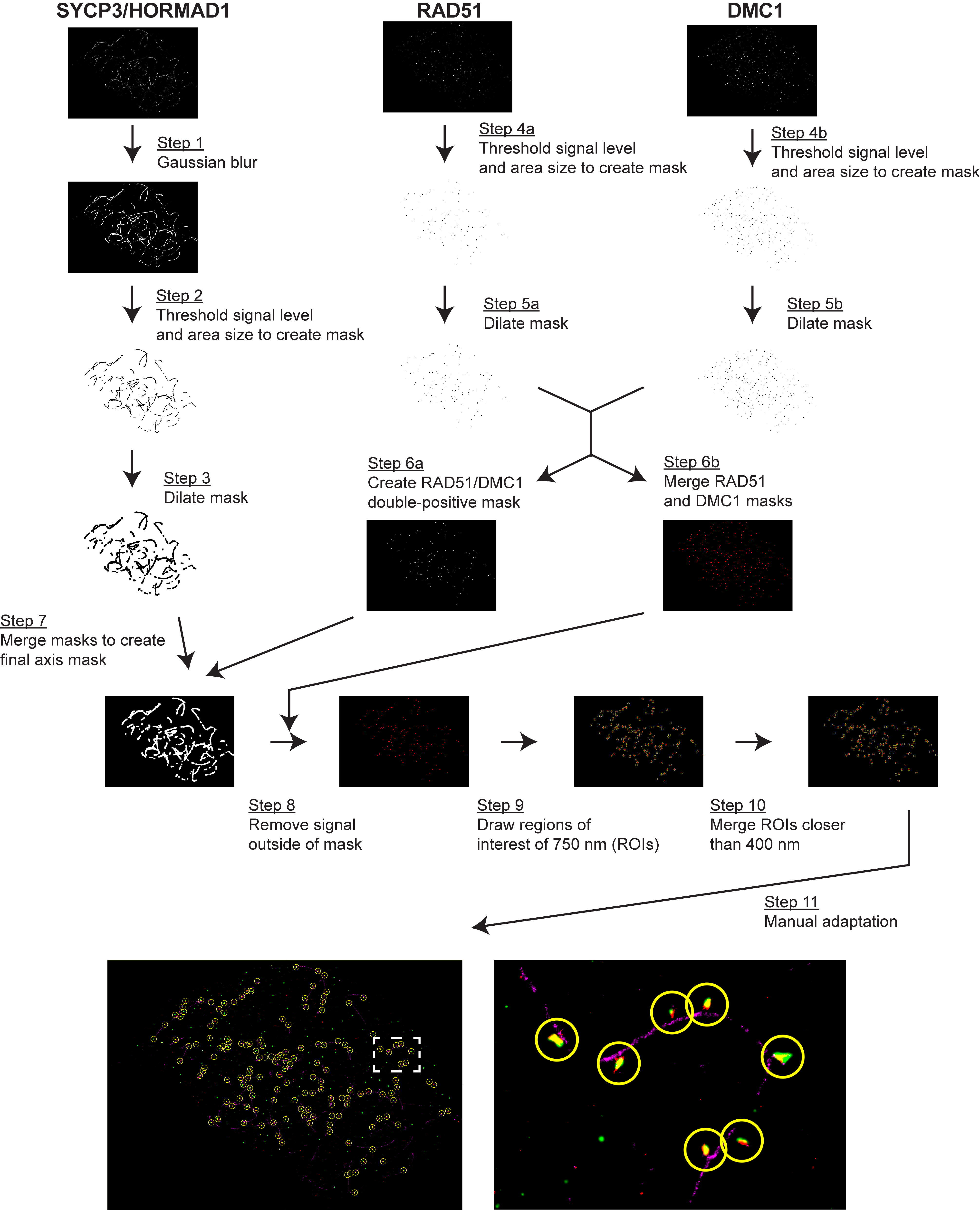

Supplement: S10 Fig — Details can be found in the material and methods. (TIF) [file pgen.1010046.s010.tif]
