# Supplementary material for: Multi-color dSTORM microscopy in Hormad1-/- spermatocytes reveals alterations in meiotic recombination intermediates and synaptonemal complex structure
Source: PLoS Genet. 2022 Jul 20;18(7):e1010046. doi: 10.1371/journal.pgen.1010046 (PMC9342782; doi:10.1371/journal.pgen.1010046)
Supplement: Attachment — Submitted filename: REVISED_Koornneefetal_responsetothereviewerscomments.docx [file pgen.1010046.s013.docx]

*Reviewer #1: This interesting manuscript reports on a study of early meiotic DNA double-strand break recognition and repair making use of super-resolution 3-color dSTORM microscopy. The authors study both WT (wild type) and Hormad1-mutant spermatocytes. The findings allow the authors to propose models for RAD51 and DMC1 recombinase configurations that may ultimately provide insight into the mechanisms of early DSB recognition. Moreover, they provide evidence that the HORMAD1 protein may inhibit formation of coils in the SC.

Overall, the manuscript is a lucid and well-written explanation of the experimental strategy and data, accessible to those interested in meiosis who may not be familiar with the imaging technology. The Introduction is generally an excellent presentation of background and strategy. It could be enhanced by adding a more compelling rationale for the selection of the Hormad1 mutant, and more clearly stating the precise questions that the research answers (e.g., what new will be resolved by the application of 3-color dSTORM, why was it not possible before, and how will the Hormad1 mutant contribute?).*

We thank the reviewer for the very positive remarks about our manuscript, both on the presentation, as well as on the experimental strategy, and the usefulness and accessibility for the meiosis research field.

As requested by this reviewer, we have now included a more specific description of the rationale for using the *Hormad1* knockout model and three-color dSTORM in the Introduction section as follows:

“The above-proposed functions of HORMAD1 in DSB induction and repair prompted us to ask whether in the absence of HORMAD1 the accumulation patterns of recombinases at meiotic DSBs would alter. In particular, we felt that the strong reduction in the number of DSBs could help to delineate how recombinase nanofoci relate to individual DSB sites. In addition, we wanted to assess whether the lifetime of certain configurations would be altered due to the loss of a possible repair-inhibiting function. Furthermore, we aimed to obtain progress in interpreting super-resolution images of recombinase accumulation in the context of SC components, using three-color dSTORM to allow more precise analyses of RAD51 and DMC1 localization patterns relative to the lateral and axial elements of the SC. In addition, using this three-color approach we could generate an independent dataset with other fluorophore combinations that allowed testing the robustness of our previous dataset, and extending the nanofocus feature set.”

*The references in this section, many of which are labeled as “recent,” are, in reality, rather old; the authors should cite some of the more recent reviews on meiotic DSB formation and repair.*

We agree that one reference which was labeled as ‘recent’ was not so very recent and we changed the sentence as follows:

“In the last decade, several studies have revealed an additional role for HORMAD1 and HORMAD2 in meiotic DSB repair (34, 46, 47).”

We also replaced the reviews cited in the paragraph of the introduction by more recent ones.

*The experimental analyses are quite clearly presented and enhanced by figures that contain both gorgeous images of the fine structure of meiotic axes and clear summary intensity distribution plots. However, one notable absent feature in imaging is the chromatin, which also is not designated in the final model figures. While it is realized that the investigators were studying DSB recognition and repair complexes, an introductory image showing chromatin background would be useful. Likewise, the addition of chromatin loops to the final models would help readers to set the overall chromosomal context, so important in consideration of homolog bias, etc.*

We agree that chromatin structure is a very important aspect of meiotic DSB formation, repair and synapsis. As the reviewer acknowledges, in our model, we zoom in on the possible DNA structures that are bound by the recombinases, and although this DNA is itself not bound by histones, we agree that it is very likely that chromatin structure is important in determining the final microscopic observations in particular in terms of localization of nanofoci relative to the SC. We have tried to incorporate this by adding a schematic representation of the chromatin loops in Figure 1K, and by adding a zoomed out schematic drawing to Figure 10, that also incorporated the SC and chromatin loops.

*As the authors acknowledge, the absence of Hormad1 results in very subtle differences in spacing and size of DxRy foci on the axes – what have we learned from these small differences about either HORMAD1 protein function or assembly of repair complexes?*

Although some of the observed changes are indeed very subtle, we feel that there is true biological significance to the observations made and we have tried to adapt the discussion section at several places to mark the key observations more clearly as follows:

“In addition, we identified new features of the nanofoci and found that although these were only subtly changed in the absence of HORMAD1, some clear functional implications arise, as detailed below.”

“If both preferred distances would generally represent distances between two ends, or between two other entities associated with a single DSB, we would expect that when less DSBs form, these two preferred distances would still be detected in similar relative frequencies. Using the *Hormad1^-/-^* model to test this hypothesis, we almost completely lost the ~900 nm preferred distance in absence of HORMAD1.”

“In absence of HORMAD1 two important changes in recombination intermediates were observed. First, we observed changes in the frequencies of the most prominent configurations that are in accordance with a possible repair-inhibitory function of HORMAD1:”

“The second key observation in *Hormad1^-/-^* spermatocytes, is the overall reduction in size of recombinase nanofoci.”

*The unexpected finding that HORMAD1 protein might negatively impact SC coiling is quite interesting. The analysis of DxRy complexes in the PAR is a great addition to this paper, but what about the Hormad1 mutant in this respect? And what about coiling along the X and Y and in the PAR?*

We thank the reviewer for these interesting suggestions. Unfortunately, we can not investigate the D_x_R_y_ complexes along the PAR in *Hormad1*^-/-^ spermatocytes since they do not reach the stage of X and Y synapsis due to the early meiotic arrest, which precludes identification of this region in the immunostainings.

Regarding coiling on and near the PAR of wild type spermatocytes, we now analyzed this in our STED images (larger number of early-pachytene nuclei compared to the dSTORM images). We found 20 suitable early-pachytene nuclei (in which the coiling of the PAR could be determined). Of those 20 regions, 9 PARs were devoid of coiling, 10 displayed a single coil and one was found with two coils. Below we provide images of several of the investigated PARs as examples; the coils are indicated by an arrowhead (scale bar represents 5 µm). Since there is only a single PAR with two coils, and the synapsed length is generally very short, it is not possible to calculate an inter-coil distance that can be compared to the inter-coil distance along the autosomes. Overall, we can conclude that the PAR appears to coil in approximately half of the nuclei, and does not display a tendency to coil more or less frequently than the autosomes. For this reason we did not incorporate this analysis in the final revised manuscript.

*Given these considerations, it is especially important that the authors more fully justify the use of Hormad1 mutants and provide a more compelling summary of precisely what has been learned from its use.*

We thank the reviewer for stressing this point, and have adapted the abstract as follows to describe this better:

“Recombinases RAD51 and its meiosis-specific paralog DMC1 accumulate on single-stranded DNA (ssDNA) of programmed DNA double strand breaks (DSBs) in meiosis. Here we used three-color dSTORM microscopy, and a mouse model with severe defects in meiotic DSB formation and synapsis (*Hormad1^-/-^*) to obtain more insight in the recombinase accumulation patterns in relation to repair progression. First, we used the known reduction in meiotic DSB frequency in *Hormad1^-/-^* spermatocytes to be able to conclude that the RAD51/DMC1 nanofoci that preferentially localize at distances of ~300 nm form within a single DSB site, whereas a second preferred distance of ~900 nm, observed only in wild type, represents inter-DSB distance. Next, we asked whether the proposed role of HORMAD1 in repair inhibition affects the RAD51/DMC1 accumulation patterns. We observed that the two most frequent recombinase configurations (1 DMC1 and 1 RAD51 nanofocus (D1R1), and D2R1) display coupled frequency dynamics over time in wild type, but were constant in the *Hormad1^-/-^* model, indicating that the lifetime of these intermediates was altered. Recombinase nanofoci were also smaller in Hormad1^-/-^ spermatocytes, consistent with changes in ssDNA length or protein accumulation. Furthermore, we established that upon synapsis, recombinase nanofoci localized closer to the synaptonemal complex (SYCP3), in both wild type and *Hormad1^-/-^* spermatocytes. Finally, the data also revealed a hitherto unknown function of HORMAD1 in inhibiting coil formation in the synaptonemal complex. SPO11 plays a similar but weaker role in coiling and SYCP1 had the opposite effect. Using this large super-resolution dataset, we propose models with the D1R1 configuration representing one DSB end containing recombinases, and the other end bound by other ssDNA binding proteins, or both ends loaded by the two recombinases, but in below-resolution proximity. This may then often evolve into D2R1, then D1R2, and finally back to D1R1, when DNA synthesis has commenced.”

*In summary this is an interesting manuscript that gives us a tantalizing taste of the power of 3-color dSTORM microscopy in elucidating the mechanisms of meiotic DSB recognition and repair. We all eagerly await the time, hopefully not too far in the future, when this kind of “nano microscopy” converges with equally revealing molecular analyses. Because the authors have already published valuable papers in this realm (refs 29 and 31), the overall impact of this contribution will be enhanced by clear statements of what is newly found and how it might change our view of recombination complexes and/or function of HORMAD1 protein. This is briefly stated in the Abstract (lines 30-32) but deserving of greater clarity and attention.*

See the changes to the abstract indicated in the previous paragraph.

*Reviewer #2: In their manuscript, Koornneef et al use three-color dSTORM to analyze the positional patterning of the recombinases, RAD51 and DMC1, in reference to axial elements in WT and Hormad1-/- mutants. Their analysis, when synthesized with prior observations from ChIP-seq data, allows the authors to provide a fascinating, albeit highly speculative, model for DMC1/RAD51 arrangement and dynamics from 3’-end resection through strand invasion during meiotic recombination. This, in combination with the truly stunning dSTORM imaging, makes this manuscript an engaging and thought-provoking read. I particularly appreciated the analysis of DMC1/RAD51 positioning on the XY (although this could have been bolstered with the use of FISH to identify the XY in all nuclei, and I wish the authors spent more time discussing the complex DMC1/RAD51 arrangements, i.e., D3R1, D3R2, etc.).*

We thank the reviewer for the encouraging and positive overall judgement of our manuscript and the microscopy methods we applied. We also appreciate the reviewers’ suggestion that RAD51/DMC1 positioning on the X and Y could also be tackled with FISH, to identify the X and Y in all nuclei. However, at present such an experiment is technically very challenging for the following reasons: To combine dSTORM and FISH, we would need an extra channel for imaging (in case of a single FISH probe), and we also would need to ensure that the FISH detection procedure would not interfere with the immunostaining. Furthermore, the PAR-probe would be ideal to recognize both X and Y with one color, but the available probe also recognize some autosomal regions, which makes identification of X and Y challenging in zygotene-stages (1).

The complex configurations indeed also deserve attention. However, they are really rare, so at this point we wish to avoid speculating too much about those. Nevertheless, we added the following to the discussion:

“We also want to note that in this model we excluded the complex configurations with more than 3 nanofoci (like D3R1). Since these configurations are quite rare, we hypothesize that they represent rare recombination intermediates which for example correspond to intermediates that are in transition from one (more frequently occurring) intermediate into the next."

*I also appreciate the use of multiple mutants (Spo11, Sycp1, Hormad2) in the evaluation of HORMAD1’s role in SC coiling. I have several reservations about this study, however, that prevent me from giving it an unequivocally glowing review:

1) The inclusion three-color dSTORM as well as the Hormad1-/- mutants allows the authors to address some important follow-up questions from their recent 2020 paper, but many of their results – particularly distance between nanofoci between WT and mutants and the positioning of DMC1/RAD51 in respect to SYCP3/HORMAD1 - seem to be very incremental developments; whether they significantly advance beyond the scope of the 2020 paper is debatable. Further, the observation that HORMAD1 appears to inhibit coiling of the SC is fairly shallow and feels like an afterthought compared to the 8 figures devoted to teasing out the fine, detailed measurements of DMC1 and RAD51 nanofoci positional data. And the differences observed between Hormad1-/- mutants and WT are so subtle, making it difficult to appreciate the biological significance of observations (e.g., marginally increased focus size in mutants). It is unclear what the broader significance of this study is (given very similar observations in the 2020 paper and decades of conventional IF staining).*

We agree that the changes observed in the *Hormad1^-/-^* model appear subtle, but we do feel that they are of biological significance. As indicated in our reply to Reviewer 1, we have now tried to explain more clearly which questions were addressed in the Introduction, and in the Discussion we now emphasized the most important advances regarding the functions of HORMAD1 and the overall interpretation of the super-resolution images. Decades of conventional IF staining showed overall colocalization of DMC1 and RAD51, and sparse individual images from EM analyses indicated that they may not be overlapping. Our 2020 paper showed for the first time that there is much variation between repair foci in the patterns of RAD51 and DMC1 accumulation and that contrary to what would be expected from recent ChIP-seq data, paired RAD51-DMC1 co-foci are rare in mouse spermatocytes. Importantly, these findings are now confirmed in the current manuscript in a completely new and very large dataset, using different fluorophores and with the possibility of adding features involving orientation of foci relative to each of the lateral elements of the SC, due to the addition of the third color in super-resolution. Furthermore, the coupling of the decrease of D1R1 with the increase of D2R1 frequencies over time, is now confirmed by the simultaneous loss of both changes over time in the mutant, confirming a functional connection between these two main conformations.

The two main novelties of this paper regarding RAD51 and DMC1 accumulation patterns are: 1) the establishment that each ROI, represents one DSB also when only one DMC1 and one RAD51 domain are observed. 2) HORMAD1 affects the lifetime of repair intermediates, consistent with faster progression of repair, as suggested by genetic data. The observed effect on coiling was indeed unexpected, but not an afterthought. We do feel that this, that the analyses of the role of synapsis (*Sycp1* mutant) and DSB repair (*Spo11* mutant), in coiling provides novel insight in the regulation of coiling of the SC.

*2) Many of the initial observations of HORMAD1 localization and DMC1/RAD51 ROI numbers are very concerning. First, the observation of HORMAD1 on synapsed regions is contrary to the generally observed trend of HORMAD1 absence along synapsed regions. Second, the number of RAD51/DMC1 ROIs observed in leptonema – zygonema (~150) seems a little low, while the number observed in pachynema (~50) is strikingly high! In C57BL/6J adult males, zygotene RAD51 foci numbers average around 250 while pachytene RAD51 foci are all but unobserved. The observation of ~50 RAD51/DMC1 ROIs in WTs is very concerning.*

We appreciate the reviewers concern and apologize if some of this may have resulted from unclarities in our manuscript. Regarding the concern about HORMAD1 localization along synapsed axes, we want to emphasize that dSTORM detects much lower signals than for example confocal microscopy. So when loss of HORMAD1 signal from synapsed axes appears rather sudden when observed with confocal microscopy, in reality there is still a significant amount of protein present in early pachytene nuclei. Moreover, in the study of Xu et al. (2) that also involves a dSTORM analysis of HORMAD1 expression, HORMAD1 signal along synapsed regions is described. In conclusion, there is no discrepancy between our results and those reported by others. We have more specifically indicated this as follows:

“Although HORMAD1 is known to be depleted from synapsed chromosome axes, this appears to be a gradual process, since with the sensitive dSTORM microscopy we still detected HORMAD1 localization on synapsed regions in early pachytene nuclei (Fig 1F-1G)(52).”

Regarding the concerns about the low ROI numbers in zygonema, we first want to stress that the numbers of DSB repair foci that can be counted using either confocal or super-resolution microscopy are determined by the method of (automated) detection. To count DSB foci in confocal microscopy, the FIJI program is often used, and depending on the settings, more foci, but also more background can be counted, or vice versa. There is a rather gradual decrease in counted foci as the settings become stricter, making setting of the cutoffs rather arbitrary. For this reason, counts of numbers of DSB foci in zygotene differ between different published manuscripts. Previously, we and others also counted around 150 RAD51 foci in zygotene nuclei (3, 4), while others detect around 200 foci (5, 6). For the dSTORM analyses, the method of ROI detection is rather strict (see for a detailed and step-by-step description of the process our response to point 9 of reviewer 3). Also, the removal of D_x_R0 and D0R_y_ caused removal of some ROIs. Finally, in a very small number of ROIs with for example a D2R1 configuration, the two DMC1 nanofoci could be at > 250 nm distance from “touching” each other (ROI diameter is 750 nm) which would resolve two foci in “standard” confocal countings. For the “high” number of ROIs in pachytenes, we apologize for not clearly indicating that these were early pachytene nuclei, and these often have this many foci, as has also been reported by others (see below in point 3). We have now indicated this more clearly throughout the revised manuscript.

*3) I am also concerned that, because most other studies rarely observe RAD51 and DMC1 in WT pachytene spermatocytes, the authors’ observations of DMC1/RAD51 nanofoci positions and dynamics in pachytene may not be illustrative of “typical” meiotic DSB repair (i.e., via interhomolog of invasion in advance of either of the two CO pathways or SDSA).*

We apologize for the apparent confusion about the pachytene-stage used in this study. Pachytene is a highly dynamic stage with regard to RAD51 and DMC1 foci. In early-pachytene stage both recombinases are still present (see for example (7, 8)), while in late-pachytene only a few RAD51 foci remain. For this study we only selected early-pachytene where both RAD51 and DMC1 foci were present. We have adapted this description throughout the manuscript.


*4) Although there is a notable decrease in the frequency of D1R1 from leptotene/early zygotene to mid-/late-zygotene (and a comparable increase in the frequency of D2R1 in the same time frame), the magnitude of these changes (particularly the latter) is ultimately very subtle – the authors’ speculation that there is a progressive evolution from D1R1 -> D2R1 -> D1R1 needs further empirical support. This proposed evolution also seems to be contradicted by the observation that D1R1 is less common in later prophase.*

We understand the reviewers concern about the progressive evolution from D1R1 to D2R1 and then back to D1R1. We think that the first transition, from D1R1 to D2R1 very likely represents subsequent steps in the repair process. This is indicated by the fact that both the reduction of D1R1 frequency, and the increase in D2R1 frequency over time are lost in the *Hormad1* knockout model, supporting the notion that these are coupled. We agree that the suggestion that, eventually, the D2R1 configuration would revert into a D1R1 configuration is at this moment not directly supported by observations, other than the finding that even in early pachytene still more than 60% of the foci are classified as D1R1. The lifetime of the different D_x_R_y_ configurations is variable, and the life time of an early D1R1 configuration, when recombinase has accumulated and the homology search is starting, might be longer than that of a late D1R1 that briefly exists during the process of DNA synthesis and recombinase removal. However, at present we agree that this is rather speculative, and have emphasized this more firmly in the revised discussion:

“When the recombinases have been completely removed from the invading strand, a D1R1 configuration may become prominent again (Fig 10B^v^). After second-end capture, or when synthesis-dependent strand annealing occurs, eventually, also the recombinases from the other ssDNA end will be removed.
 With this speculative model, we present a plausible relationship between the most frequently observed D_x_R_y_ configurations and corresponding loading pattern of recombinases on the ssDNA.”

*5) The authors’ analysis of D2R1 orientation around the SC showed a minority of RAD51 inside of lateral elements; however, the authors conclude that this supports a model for a D2R1 dumbbell structure with RAD51 located in the center of the SC – this is just not born out by the majority of the data.*

We apologize for this confusion. We agree that the center of mass of 60% of the RAD51 foci is still localized outside the lateral elements. However, part of the RAD51 nanofocus in this structure is still often localized between the two lateral elements, this can be inferred from the rotation analyses that shows that the RAD51 signal is localized in the center of the SYCP3 signal in the summed image. To more precisely explain the situation we have adapted the text as follows:

“This analysis showed that overall, the center of mass of the nanofoci was positioned more frequently outside the lateral elements than in between. Of all the nanofoci, RAD51 of D2R1 is most frequently positioned between the two lateral elements (Fig 7H), which is in accordance with the observed distances to the SC and the results of the rotation analyses (Fig 7A). Thus, these data again argue for a D2R1 dumbbell structure with RAD51 located nearest to the center of the SC and the two DMC1 nanofoci located on each side, further away from the center.”

*6) I want to stress that I appreciate the amount of work the authors put into image acquisition and analysis. Although the total number of ROIs analyzed was high, the number of biological replicates was shockingly low – only three animals analyzed per genotype, with approximately 10 nuclei or fewer analyzed per prophase stage per animal.*

We thank reviewer 2 for the appreciation for the amount of work. However, we respectfully disagree that the number of nuclei and biological replicates is low. Previous dSTORM studies which mainly focus on the synaptonemal complex do not mention the number of analyzed chromosomes, let alone the number of analyzed nuclei or biological replicates (2, 9). Studies which do mention numbers of analyzes chromosomes use between 4-94 which can be analyzed from around 5 nuclei (10, 11). Thus, our study where we analyzed 91 nuclei in total is enormous compared to other meiotic dSTORM studies. In confocal analyses of foci numbers, the number of biological replicates is also usually 2 or 3 (e.g. (12, 13)), sometimes only the number of analyzed nuclei is reported, and not the number of mice (14). Finally, we have assessed each mouse dataset separately, and observed no statistically different features between mice of the same genotype (one-way ANOVA test on the D1R1 category in wild type revealed a p-value of 0.364 and one-way ANOVA test on the D1R1 category in the knockout revealed a p-value of 0.085). Note that for wild type 4 different animals were used (3 different animals for the HORMAD1-RAD51-DMC1 dataset and 3 different animals for SYCP3-RAD51-DMC1, but when combined this results in 4 different animals).

*7) Distinctions of what constitutes a single focus are a little nebulous. For instance, D2R1 configurations have one DMC1 focus much smaller than the other, with an elongated/oblong RAD51 focus. Is it possible the oblong “focus” is actually two foci that are too close to be resolved by dSTORM? With this in mind, it is difficult to discern what variation in focus size and shape mean in term of number of whether the focus is “real” or nonspecific/background staining.*

We apologize that our descriptions of how (nano) foci were detected was not fully clear. We have now included a step-by-step description of the method that we used to automatically detect foci as well as nanofoci, and how we tried to reduce background as much as possible (see reviewer 3 point 9). Obviously, the resolution is limited by the sizes of the primary and secondary antibodies, which is expected to add around 20–40 nm in X and Y direction in our 2D images. In addition, the precision of each localization event varies, and affects the Gaussian distribution-based representation of the dSTORM data. In addition, variability in antibody binding may influence the actual “shape” generated by the Gaussian distribution generated from the localization events coming from the fluorophores attached to the secondary antibodies. Taking all this into account, we feel that the use of binary images provides an optimal representation of the actual areas covered by the recombinases and bound antibodies; it reduces complexity but also corrects for artifactual irregularities.

*8) It is critical throughout the manuscript to be clear about what stage is being analyzed – at several points throughout I was uncertain whether the authors were discussing leptotene or early-, mid- or late-zygotene. Similarly, it is critical the authors specify what mouse strain the WT and mutants are on.*

We thank the reviewer for pointing at this ambiguity regarding the stage of nuclei in the text. During the revision, we have explicitly stated which stages were analyzed at appropriate places and hope that this is now resolved.

In the materials and methods section, we now also specified the mouse strain background of the mice used in this study*.*

*Reviewer #3: This manuscript by Koornneef et al. reported dSTORM imaging of RAD51, DMC1 and axial/lateral elements (SYCP3) in mouse spermatocytes. By comparing wildtype and Hormad1-/- knockout mice, the authors detected some minor changes of DMC1-RAD51 configurations in the absence of HORMAD1. Integrating recent findings using RAD51 and DMC1 ChIP-seq in mice, authors proposed a revised model to interpret microscopic images (some modifications compared to their previous model) and accommodated major DxRy configurations into recombination pathway.

In general, the results shown here provide new insights into how RAD51 and DMC1 are possibly organized at DSB sites. Yet, it is a bit disappointing that these results cannot explain HORMAD1 functions nor directly understand more about how HORMAD1 affects DMC1-RAD51 patterns and SC coiling.

Having said that, this is an interesting story. My main concern is that the revised model to explain DMC1-RAD51 configuration is partially based on comparisons of distances/distribution/numbers of ROI and nanofoci in wild type and Hormad1-/- knockout mice. However, without knowing the distribution of decreased DSBs in mutants, whether HORMAD1 is involved in repair pathway choice and whether HORMAD1 affects DSB end processing, it seems to be shaky to use their patterns of DMC1-RAD51 to explain DMC1-RAD51 configurations in wild type. This concern, along with other more minor comments and suggestions for the manuscript are listed below.*

We thank Reviewer 3 for the overall positive comments on our manuscript regarding new insights in recombinase loading, and the overall interesting story. We hope that the concerns raised by this reviewer will be resolved through our detailed response to the points below.

*1. Abstract is not well summarized. Seemingly, the study aims to understand how recombinases are loaded on ssDNA in the absence of HORMAD1. However, I think results have little information that can link DMC1-RAD51 patterns to HORMAD1 functions.*

We have rewritten the abstract to better indicate the implications of our findings in general, and in relation to HORMAD1 functions.

*2. I think the most important results of this study can be the modification of previous interpretation of DMC1-RAD51 configurations on each end of DSBs, but it may require supporting evidence of other mutants that are directly defective in DSB processing or repair.*

We agree that one of the important conclusions of the manuscript is that a D1R1 configuration represents loading on one DSB. Whether this then represents loading on one end of such a DSB requires further study, and we agree that DSB-processing mutants would be a good next model to analyze. However, we feel it is outside the context of the current manuscript to be able to ascertain whether D1R1 represents two ends close together, or one end, since multiple mutants, and multiple combinations of antigen detections will be required. For example, we are now also studying accumulation of ssDNA binding proteins such as RPA in combination with the recombinases, and mutants in which recombinase loading is impaired or absent.

*3. Line 109-110 states that Hormad1 -/- leads to meiotic arrest at zygotene. But later, analysis used pachytene-like stages for mutants. Perhaps, adding more description to clarify how staging was performed for mutant and/or rephrasing “meiotic arrest at zygotene”.*

We apologize for the unclarities in our description of the arrest stage of the *Hormad1^-/-^* model. In the original knockout paper, TUNEL analyses of testes sections showed that cells are eliminated between Stage IV and V. At this stage of the spermatogenic cycle, mid-pachytene spermatocytes should be present. However, the cells in this mutant, and in many other mutants with this “pachytene arrest” phenotype never reach full synapsis of all chromosomes, so the appearance in immunocytochemical stainings of the SC is more like a zygotene nucleus. Still, based on the current knowledge concerning the functional connection between failure of XY body formation (which occurs in pachytene) and the stage IV arrest phenotype, it is generally accepted that the timepoint of development of the furthest developed cell-type corresponds to pachytene, and the cells are then referred to as zygotene-pachytene or pachytene-like. We now refer to this stage as early pachytene-like throughout the revised manuscript and have adapted the text of the introduction as follows:

“Deficiency in *Hormad1* leads to infertility in both sexes, reduction of recombination foci numbers of RAD51, DMC1, and RPA, and meiotic arrest at an early pachytene-like stage, but with incomplete chromosome synapsis (33-35). This phenotype is also known as a Stage IV arrest, that refers to the stage of the spermatogenic cycle at which spermatocytes should have reached mid-pachytene, but are eliminated by apoptosis, triggered by failure of XY body formation (36-39).”

*4. Lines 122-126 and also lines 527-531, studies mentioned here were performed in spo11-deficient backgrounds, where DSBs are exogenously induced and ssDNA processing and repairing may not be canonical, so it may be inappropriate to use these findings to explain DMC1-RAD51 patterns observed in Hormad1-/-.*

We have explained more clearly in the Introduction that we refer to damage-induced DSBs, but also included information suggesting that they are progressed in a manner similar to meiotic DSBs, and in the Discussion we added an extra sentence as follows:

Introduction:

“Although it should be noted that the DSBs analyzed were radiation-induced, both RAD51 and DMC1 accumulate at such DSBs, they (re)localize to the SC, and contribute to homology recognition (50). Also, Cisplatin-induced DSBs were shown to contribute to homologous chromosome synapsis in a hybrid sterility mouse model (51).”

Discussion:

“This fits with the results of a study in *Hormad1^-/-^Spo11^-/-^* spermatocytes where exogenous induced DSBs were repaired faster (49). Our data now provide the first indication that HORMAD1 may similarly affect processing of endogenous SPO11-induced DSBs.”

*5. Lines 148-151, please describe the analysis/sampling of randomized control in Material and Methods.*

We have replaced the brief description of this method by a more detailed explanation as follows:

“To generate a random control of HORMAD1 localization along the axial elements, an unsynapsed region of a chromosomal axis (shown in Figure 1I) was chosen. After measuring the intensity profile as described above, we first determined the boundary of the localization events, to create a mask. Next, we took the number of localization events that were within this mask, to generate a new random pattern with random precisions per localization event in the range of the measured localizations. We generated 50 such random patterns within this mask. The random intensity plot that is shown in Figure 1I is the average of all the 50 line profiles of the 50 generated random localization event patterns”

*6. Lines 155-156 state that two HORMAD1 rods run along each side of the axial elements, and also in the same plane with SYCP1. To support this, please show a representative “3D-dSTORM” of HORMAD1 and SYCP1 in frontal and axial views.*

We understand that visualization of SYCP1 together with HORMAD1 in 3D-dSTORM would support our statement that HORMAD1 runs in the same plane as SYCP1. However, at present, performing a two-color 3D-dSTORM is still a very technically challenging technique. In particular, the correction of x, y and z drift and the alignment of two channels are still not well-enough optimized for us to generate reproducible and interpretable 2-color 3D-dSTORM. Therefore, we chose to perform one-color 3D-dSTORM. Moreover, as written in the manuscript, we performed this 3D-dSTORM experiment to see if these double axes were also present in a 3D-enviroment. As shown in Fig 1J HORMAD1 localization can be seen as double axes in both X-Y as Y-Z directions. This, in combination with the 2D-dSTORM of HORMAD1 and SYCP1 where you clearly see that when SYCP1 signal can be resolved as two axes, HORMAD1 patterns are resolved as 4 axes, strongly suggest a situation as outlined in the text and Fig 1K.

*7. Please show original images if acquired by widefield microscopy, as the Fig 1A has traces of background cropping.*

We replaced the widefield images in Fig 1A and Fig 1E with the original images. We indicated the region which was imaged with dSTORM microscopy. We also changed this in the legend of this figure.

We also corrected a typographical error in the description of scale bar (represents 5 µm, not 1 µm) of Figure 1 A,B, E and F.

*8. Please provide supporting data or references to demonstrate specificity of RAD51 and DMC1 antibodies used in this study.*

At present, we cannot unequivocally assess the specificity of both antibodies, due to lack of an appropriate RAD51-negative meiotic cell type. To approximate this situation as best as possible we have performed immunostainings on *Dmc1* knockout slides to verify specificity of DMC1 and maintenance of RAD51 signal on these slides. In addition, we used *Spo11* knockout slides to show loss of almost all recombinase staining in this mode (a few naturally occurring damage-induced DSBs are always present, see also our previous analyses of recombinase foci in the *Spo11* knockin null mutation model (3)). We have added the results to a new supplemental Figure 9 and described the experiments in the Materials and Methods sections as follows:

“Control immunostainings in *Dmc1^-/-^* and *Spo11^-/-^* spermatocytes, which were imaged using a Zeiss Confocal Laser Scanning Microscope 700 with a 63x objective immersed in oil (images were taken with the same intensity), confirmed loss of DMC1 signal, and loss of signal of both recombinases, respectively (S9 Fig).”

*9. Lines 168-170, for this study and conclusion made later, selection of ROIs is critical. Please provide a supplemental figure to demonstrate procedures step-by-step of recombination foci analysis for lines 711-727.*

We appreciate this suggestion and generated a new Supplemental Figure 10 that provides such a step-by-step demonstration of the method.

*10. Since size of ROIs may change how the authors interpret DMC1-RAD51 configurations, can authors track axes or SCs and measure distances of associated DMC1-RAD51 on each axis/SC? It should confirm the 800-900 nm distance. Also it will be helpful to count how many associated DMC1-RAD51 configurations in wild type and mutants to support ROIs being correctly assigned.*

In the procedure to select ROIs there is a step in which we create a dilated mask of the axis/SC and only include signals with a center of mass that is within the mask, to only select axis-associated nanofoci. So all analyzed ROIs in both wild type and *Hormad1^-/-^* spermatocytes are axis associated. Indeed, in our nearest neighbor analyses, we did not take the track along the SC into account. We have now performed such an analysis using the distances between subsequent coordinates along the manually drawn axes. The coordinates correspond to the intersection of the line from the center of mass from each nanofocus to the nearest manually drawn axis. The results are very similar to those that were obtained with the nearest neighbor analysis without the axis taken into account. Both preferred distances were observed, with a small shift of the peak around ~300 nm towards smaller distances. This could be explained by the method that calculated the distance between coordinates along the axis lines, instead of the distance between center of mass of nanofoci, resulting in shorter distances for example for the two DMC1 nanofoci in a D2R1 configuration. In addition, in this new analysis, we also observed a decrease in frequency of the preferred distance around ~900 nm in the *Hormad1^-/-^* spermatocytes. We want to note that this decrease is less outspoken compared to what was observed in the nearest neighbor analysis without axis tracking. This may be partially explained by the fact that single ROIs on an axes fragment were excluded from analysis, and this occurs relatively more frequently when the total number of ROIs is lower, such as in the *Hormad1* knockout. This leads to an overrepresentation of the data which remains included. The remaining very small 900 nm peak may also indicate that even in the *Hormad1* knockout there is a certain degree of even spacing of meiotic DSBs along hotspot-rich regions of certain chromosome axes. We added the following to the text:

Results:

“A limitation of this analysis is that the position of ROIs along the synaptonemal complex was not taken into account, which could lead to a different distribution. Therefore, we performed an additional analysis using the manually drawn axes in zygotene and early pachytene nuclei. We defined coordinates along these lines by determining the intersection between a line drawn from the center of mass from each nanofocus to the nearest SC or axial element. The frequency distribution of nearest neighbor distances between these coordinates along the tracked axes showed a very similar distribution to that of the nearest neighbor analysis of nanofoci only, although a small peak around 750-1050 nm could still be discerned (S3 Fig). This may in part be due to the fact that more data points are lost in this type of analysis (axes that have only a single ROI associated with it are more frequent in the knockout, and are excluded here), but may also reveal some remaining organized distribution of DSBs in hotspot-rich regions.”

Material and methods:

“For the nearest neighbor analysis where the axes were taken into account, the following strategy was used. First, the coordinate on the manually drawn axis line closest to the nanofocus was determined for all nanofoci. Next, the nearest neighbor of these coordinates was examined and the distance between the pair (along the manually drawn line) was calculated. The manually drawn lines (for details see section about the distance to the axes) follow the SYCP3 signal, where a distinction was made between unsynapsed and synapsed regions. For synapsed regions, a single line between the two SYCP3 axes was drawn. We excluded single ROIs on an axes fragment, since there are only intra-ROI nanofoci distances in such an instance. We also excluded leptotene and leptotene-like nuclei from this analysis.”

*11. The authors can consider presenting distribution of nearest neighbor analysis of nanofoci first. Along the analysis of tracing axes or SCs, these results may justify parameters of ROI selection. So, the notion that number of ROIs in wild type and mutants are similar to their DSB numbers is more convincing. It also can support the proposed model in which each configuration represents one DSB (both ends of a DSB), rather than one end of a DSB.*

We agree that changing this order may help to make the point that each configuration within a ROI represents a DSB. However, as is now more clearly explained in S10 Figure, the selection of nanofoci follows after the selection of ROIs in the procedure. This complicates implementing a description of nanofoci before describing the ROI selection. For this reason, we kept the order as it was.

*12. Please show an additional real image (together with SYCP3 signals) for Fig 2O.*

We added a real image with SYCP3 signals of wild type to Fig 2O. We also indicated the origin of the lines as described by point 13.

*13. Need better description for Fig 2O, such as double lines vs single lines.*

We adapted the figure to better indicate the origin of the lines by changing these into arrows and have adapted the legend for Fig 2O as follows:

“DMC1 nanofoci are depicted in green and RAD51 nanofoci in red. Dashed circles indicate a ROI and for every DMC1 nanofocus the shortest distance to the nearest DMC1 neighbor is indicated by a line with an arrow.”

*14. Lines 197-200, if the reduction of DSB numbers in Hormad1-/- knockout spermatocytes is not uniform, the lower frequency in 750-1050 nm may not reflect reduced DSB numbers in mutants. So, I suggest additional analysis to track axes or SCs in wild type and mutants at early stage.*

As indicated in our reply to point 10, we have implemented this track-axis nearest neighbor analysis in our revised manuscript.

*15. Lines 270-273 and Fig 4C. In addition to the eccentricity, analyzing “Feret” may provide significant difference their shapes.*

In accordance with the reviewers’ suggestion, we have also performed an analysis of the Feret parameter. We measured the eccentricity based on the maximal and minimal Feret values for each nanofocus (instead of the eccentricity in the manuscript which was based on the minor and major axis of an fitted ellipse) to assess changes in eccentricity, since a longer or shorter maximal Feret value alone, may be explained by changes in size, not shape. The results are shown below and correspond well to our analyses of eccentricity. Similar to the eccentricity in the manuscript, no significant differences were found between wild type and knockout for the Feret eccentricity. Therefore, we decided not to include these data in the present manuscript.

*17. Line 377 and Fig 7H, nanofoci outside of lateral elements include signals in between lateral elements (inside of the SC)? I assume authors refer to signals outside of SC?*

Indeed, we meant refer to a signal (center of mass) in between lateral elements versus signal outside the SC. We have corrected the description in the legend of the figure and provided example images to make the qualifications clearer*.*

*18. Which configuration is most likely to form CO? D2R1 or D1R2? Or is there any, even more complex configurations that may possibly represent CO designation?*

At present we do not know whether there are specific recombinase accumulation patterns that would be predictive of a (future) crossover site. The best known crossover marker, MLH1 appears when recombinases have already disappeared. MSH4, which partially colocalizes with MLH1 in mid- and late pachytene nuclei, may be involved in stabilizing Holiday junctions and appears somewhat earlier. In future experiments it may be worthwhile to assess MSH4 signals in combination with one or both recombinases, but at present we have no good MSH4 antibody that could be used for this type of experiment. From the data in this manuscript, it seems that the D2R1 configuration may represent a substage in DSBs that become COs, also because this configuration is enriched on the PAR. However, it is equally likely that it could be a substage for crossover as well as noncrossover end-points.

*19. The D1R2 number seems to be consistent across stages, are they similar to CO numbers per meiosis in mouse?*

Indeed, the D1R2 configuration frequency doesn’t change during the meiotic prophase, but since there are more ROIs in the early stages, compared to the later stages, the absolute number does change; the mean value of the number of D1R2 foci decreases from 8 in leptotene to 3 in pachytene, so this is much lower than the 21-27 crossovers that can be detected in each nucleus. However, how many foci with a certain recombinase configuration can be observed at a certain stage also depends on the lifetime of that intermediate, so it does not exclude the possibility that a D1R2 configuration (also) accompanies crossover formation.

*20. Please deposit original images in public data repository.*

The original super-resolution images are now stored on BioImage Archive and will be available when this manuscript is published. The reviewers can access the data already via this link to see the raw images before publication: [https://eur01.safelinks.protection.outlook.com/?url=https%3A%2F%2Fwww.ebi.ac.uk%2Fbiostudies%2Fstudies%2FS-BIAD423%3Fkey%3Da157f22d-3d26-4dfe-8082-8f9453581f2e&amp;data=04%7C01%7Cw.baarends%40erasmusmc.nl%7C5d94a0abfbc94a44f67608da1ba929ef%7C526638ba6af34b0fa532a1a511f4ac80%7C0%7C0%7C637852710602869378%7CUnknown%7CTWFpbGZsb3d8eyJWIjoiMC4wLjAwMDAiLCJQIjoiV2luMzIiLCJBTiI6Ik1haWwiLCJXVCI6Mn0%3D%7C3000&amp;sdata=tmH1CUX4BEdlhMHJtIpOiEKe%2FL8FfA0a%2FSEslGueS2w%3D&amp;reserved=0](https://eur01.safelinks.protection.outlook.com/?url=https%3A%2F%2Fwww.ebi.ac.uk%2Fbiostudies%2Fstudies%2FS-BIAD423%3Fkey%3Da157f22d-3d26-4dfe-8082-8f9453581f2e&data=04%7C01%7Cl.koornneef%40erasmusmc.nl%7Cd8b6ff15e1224c9e47dd08da1c5032ac%7C526638ba6af34b0fa532a1a511f4ac80%7C0%7C0%7C637853428026238423%7CUnknown%7CTWFpbGZsb3d8eyJWIjoiMC4wLjAwMDAiLCJQIjoiV2luMzIiLCJBTiI6Ik1haWwiLCJXVCI6Mn0%3D%7C3000&sdata=drSAHpiovVqEtqIYlo2%2FQ%2BdY0ZQOwHbjVBK3oklUSeo%3D&reserved=0)

We added the following to the manuscript.

“**Data availability**

The microscopic data generated in this study (dSTORM and STED) have been deposited in BioImage Archive (86) in the BioStudies (87) database under accession code S-BIAD423 [https://www.ebi.ac.uk/biostudies/studies/S-BIAD423].”

**References:**

1.	Acquaviva L, Boekhout M, Karasu ME, Brick K, Pratto F, Li T, et al. Ensuring meiotic DNA break formation in the mouse pseudoautosomal region. Nature. 2020;582(7812):426-31.

2.	Xu H, Tong Z, Ye Q, Sun T, Hong Z, Zhang L, et al. Molecular organization of mammalian meiotic chromosome axis revealed by expansion STORM microscopy. Proc Natl Acad Sci U S A. 2019;116(37):18423-8.

3.	Carofiglio F, Inagaki A, de Vries S, Wassenaar E, Schoenmakers S, Vermeulen C, et al. SPO11-independent DNA repair foci and their role in meiotic silencing. PLoS Genet. 2013;9(6):e1003538.

4.	Ishishita S, Matsuda Y, Kitada K. Genetic evidence suggests that Spata22 is required for the maintenance of Rad51 foci in mammalian meiosis. Sci Rep. 2014;4:6148.

5.	Dai J, Voloshin O, Potapova S, Camerini-Otero RD. Meiotic Knockdown and Complementation Reveals Essential Role of RAD51 in Mouse Spermatogenesis. Cell Rep. 2017;18(6):1383-94.

6.	Kauppi L, Barchi M, Lange J, Baudat F, Jasin M, Keeney S. Numerical constraints and feedback control of double-strand breaks in mouse meiosis. Genes Dev. 2013;27(8):873-86.

7.	Li M, Feng H, Lin Z, Zheng J, Liu D, Guo R, et al. The novel male meiosis recombination regulator coordinates the progression of meiosis prophase I. J Genet Genomics. 2020;47(8):451-65.

8.	Shang Y, Huang T, Liu H, Liu Y, Liang H, Yu X, et al. MEIOK21: a new component of meiotic recombination bridges required for spermatogenesis. Nucleic Acids Res. 2020;48(12):6624-39.

9.	Prakash K, Fournier D, Redl S, Best G, Borsos M, Tiwari VK, et al. Superresolution imaging reveals structurally distinct periodic patterns of chromatin along pachytene chromosomes. Proc Natl Acad Sci U S A. 2015;112(47):14635-40.

10.	Agostinho A, Kouznetsova A, Hernandez-Hernandez A, Bernhem K, Blom H, Brismar H, et al. Sexual dimorphism in the width of the mouse synaptonemal complex. J Cell Sci. 2018;131(5).

11.	Schucker K, Holm T, Franke C, Sauer M, Benavente R. Elucidation of synaptonemal complex organization by super-resolution imaging with isotropic resolution. Proc Natl Acad Sci U S A. 2015;112(7):2029-33.

12.	Felipe-Medina N, Caburet S, Sanchez-Saez F, Condezo YB, de Rooij DG, Gomez HL, et al. A missense in HSF2BP causing primary ovarian insufficiency affects meiotic recombination by its novel interactor C19ORF57/BRME1. Elife. 2020;9.

13.	Prakash R, Sandoval T, Morati F, Zagelbaum JA, Lim PX, White T, et al. Distinct pathways of homologous recombination controlled by the SWS1-SWSAP1-SPIDR complex. Nat Commun. 2021;12(1):4255.

14.	Zhang Q, Ji SY, Busayavalasa K, Shao J, Yu C. Meiosis I progression in spermatogenesis requires a type of testis-specific 20S core proteasome. Nat Commun. 2019;10(1):3387.
